# CHRONIC DISEASE AND TERMINAL DECLINE IN VERY OLD MEN: THE MANITOBA FOLLOW-UP STUDY

**DOI:** 10.1093/geroni/igz038.485

**Published:** 2019-11-08

**Authors:** Robert B Tate, Philip St John, Audrey Swift, Edward H Thompson

**Affiliations:** 1 University of Manitoba, Winnipeg, Manitoba, Canada; 2 University of Manitoba, Winnipeg, Canada; 3 College of the Holy Cross, Worcester, Massachusetts, United States

## Abstract

The Manitoba Follow-up Study is in its 71st year of continuous operation. Since 1948, 3,983 aircrew recruits to the Royal Canadian Air Force during the Second World War have submitted routine medical examinations and completed questionnaires. On May 1, 2006, 1001 of these men (25%) were alive mean age of 86 years. The effects of 7 chronic diseases (CDs) diagnosed before 2006 were modeled with multinomial logistic regression to predict the pattern of decline of living and dying through an 11 year window to 2017. By 2017, 11% were still alive, 10% died very early in the window, 44% experienced a slow decline of a least three years to death, 17% experienced a step decline to death, and 18% experienced a terminal drop, death within six months of decline in functioning. Only 30% were free of CD in 2006; 36% had 1 CD, and 34% had more than 1 CD. As the number of CDs increased, the probability of remaining alive by 2017 decreased: 18% alive if no CD, 10% if 1 CD, 8% if 2 CDs, and 3% of >2 CDs. The chance of death with a terminal drop decreased: 22% if no CD, 20% if 1 CD, 14% if 2 CD, 11% if >2 CD. Conversely, the percent with a gradual decline to death increased with more CDs. Among old men are already in their 80s, a key determinant of the trajectory to end of life is the number of CDs.

